# *R*-Roscovitine (Seliciclib) prevents DNA damage-induced cyclin A1 upregulation and hinders non-homologous end-joining (NHEJ) DNA repair

**DOI:** 10.1186/1476-4598-9-208

**Published:** 2010-08-04

**Authors:** Mario Federico, Catherine E Symonds, Luigi Bagella, Flavio Rizzolio, Daniele Fanale, Antonio Russo, Antonio Giordano

**Affiliations:** 1 Sbarro Health Research Organization, Center for Biotechnology, College of Science and Technology, Temple University, Philadelphia, Pennsylvania, USA; 2 Department of Surgery and Oncology, University of Palermo, Palermo, Italy; 3 Division of Biochemistry and Biophysics, Department of Biomedical Sciences, National Institute of Biostructures and Biosystems, University of Sassari, Sassari, Italy; 4 Program in Genetic Oncology, Department of Human Pathology and Oncology, University of Siena, Siena, Italy; 5 Dipartmento Discipline Chirurgiche ed Oncologiche, sezione di Oncologia Medica, Policlinico Universitario Paolo Giaccone, via del Vespro 127, 90127, Palermo Italy; 6 SHRO, Bio-life Sciences Building Suite 400, 1900 North 12th St., Philadelphia, PA 19122, USA

## Abstract

**Background:**

CDK-inhibitors can diminish transcriptional levels of cell cycle-related cyclins through the inhibition of E2F family members and CDK7 and 9. Cyclin A1, an E2F-independent cyclin, is strongly upregulated under genotoxic conditions and functionally was shown to increase NHEJ activity. Cyclin A1 outcompetes with cyclin A2 for CDK2 binding, possibly redirecting its activity towards DNA repair. To see if we could therapeutically block this switch, we analyzed the effects of the CDK-inhibitor *R*-Roscovitine on the expression levels of cyclin A1 under genotoxic stress and observed subsequent DNA damage and repair mechanisms.

**Results:**

We found that *R*-Roscovitine alone was unable to alter cyclin A1 transcriptional levels, however it was able to reduce protein expression through a proteosome-dependent mechanism. When combined with DNA damaging agents, *R*-Roscovitine was able to prevent the DNA damage-induced upregulation of cyclin A1 on a transcriptional and post-transcriptional level. This, moreover resulted in a significant decrease in non-homologous end-joining (NHEJ) paired with an increase in DNA DSBs and overall DNA damage over time. Furthermore, microarray analysis demonstrated that *R*-Roscovitine affected DNA repair mechanisms in a more global fashion.

**Conclusions:**

Our data reveal a new mechanism of action for *R*-Roscovitine on DNA repair through the inhibition of the molecular switch between cyclin A family members under genotoxic conditions resulting in reduced NHEJ capability.

## Background

The cell cycle is comprised of a series of highly coordinated events culminating in cell growth and division. Cyclin-dependent kinases (CDK) and their cyclin counterparts strictly regulate and drive cell cycle progression and different CDK/cyclin complexes are responsible for the timely occurrence of each phase transition in order to maintain genetic integrity throughout generations. Cancer cells have been frequently found to have a de-regulated CDK activity allowing them to escape the normal cell cycle and proliferate uncontrollably. For these reasons CDKs have been considered attractive targets for cancer therapy and several CDK-inhibitors have been developed and are under intense investigation[1].

*R*-Roscovitine (Seliciclib, CYC202; herein referred to as Roscovitine), one of the most promising members of the CDK-inhibitor family, is an orally available adenosine analogue prominently targeting CDK2 (also affecting CDKs 1, 7 and 9 at a much lower rate)[2] with a low off-target effect on other members of the human kinome[3] , and a nice toxicity profile[4]. In preclinical studies Roscovitine has shown significant *in vitro *and *in vivo *antitumor activity on a wide panel of human cancers and is currently in phase II clinical trials[5]. Since preclinical experimentation, it has become evident that, CDK-inhibitors, such as Roscovitine, may actually curb the activity of DNA repair machinery[6,7], hence becoming an attractive candidate for therapeutic association with either radiation therapy[8,9] or genotoxic agent-based chemotherapy[10]. However, the mechanism of this inhibition is still elusive.

One of the proposed means for CDK-inhibitors to affect DNA repair is through checkpoint deregulation[11-13], but increasing evidence supports a complex network of direct interactions between individual CDKs and proteins that play a key role in DNA damage repair (DDR). It is known that different DNA repair pathways are preferentially activated at specific stages of the cell cycle possibly suggesting a functional crosstalk between CDK/cyclin complexes and DNA repair mechanisms[14]. In particular, CDK2 has been shown to interact with p53[15], BRCA1[16], BRCA2[17], Ku70[18] and both, CDK1 and CDK2, can modulate BRCA1-BARD1 activity[13,19]. Moreover, CDK2 knock-down cells have an attenuated capacity to repair DNA damage suggesting a pivotal role for CDK2[7] in DDR. Given the ability of CDKs to compensate for each other *in vivo*, overall CDK activity has been proposed to be influential in DDR regulation[20] however CDK2 function seems to have a specific role in some survival pathways[21].

Cyclins, similarly to CDKs, have been correlated to DDR. Cyclin E levels are upregulated under genotoxic stress conditions[22] and a post-translational cleavage generates an 18-amino acid peptide, which has been shown to interact with Ku70[18] promoting the release of the pro-apoptotic factor Bax from the inactivating complex Bax/Ku70. Moreover, an increasing amount of data suggests an important role in DDR for the A-type cyclins, and in particular for cyclin A1. Differing from cyclin A2, ubiquitously expressed during the S and G2/M phases of the cell cycle, cyclin A1 is a testis-specific cyclin, which interacts with CDK2 and is involved in germ cell meiosis and spermatogenesis[23]. Cyclin A1 may have a role in carcinogenesis, as it has been found to be over-expressed in acute myeloid leukemia and various other tumour types[23-25], however, its role in cancer is still particularly obscure. In somatic non-testicular tissues, cyclin A1 is not expressed or is expressed at very low basal levels. After genotoxic insult, cyclin A1 mRNA is upregulated *in vitro*[26] and *in vivo*[27]. At a molecular level, human CDK2/cyclin A1 complexes interact with members of the Ku family and phosphorylate Ku70[27,28], a pivotal player in the non-homologous end-joining (NHEJ) double strand break (DSB) repair pathway. Furthermore, under genotoxic conditions the kinase activity of CDK2/cyclin A1 complex increases, while the relative kinase activity of CDK2/cyclin A2 decreases and the CDK2/cyclin A1 complex out-competes with CDK2/cyclin A2 for Ku70 binding[28]. Moreover, it has recently been found that CDK2 phosphorylation status and structure changes upon the cyclin A family member with which it is bound [29] suggesting a non-redundant function between CDK2/cyclin A1 and CDK2/cyclin A2 complexes. Finally cyclin A1 knockout mice and *Xenopus *embryos exhibited a clear defect in DNA repair[27,30] and are more prone to undergo apoptosis[31].

Taken together these data support that during genotoxic stress differential transcriptional levels and activity of cyclin A family members may redirect CDK2 toward DNA repair resulting in a modulation of NHEJ. Since one of the most relevant effects of CDK inhibitors is the downregulation of cell cycle related cyclins, we investigated if the inhibition of DNA repair mechanisms by Roscovitine may also occur through the modulation of the expression levels of cyclin A family members. Physiological CDK-inhibition, in fact, results in cyclin downregulation through the inhibition of E2F-family transcription factors, which drive and regulate cell cycle-related cyclin transcription. Given that the promoter of the cyclin A1 gene, *CCNA1*, is different from the other cell cycle-related cyclins, not being under the regulation of E2Fs[32], here we analyzed the effects of Roscovitine on cyclin A1 expression and modulation of DNA repair mechanisms. We demonstrated that under DNA damaging conditions cyclin A1 is strongly upregulated and localizes to the nucleus. Although Roscovitine alone was not sufficient to reduce the basal levels of cyclin A1, in contrast to cell cycle related cyclins, Roscovitine treatment could abolish the DNA damage-induced cyclin A1 upregulation, reducing NHEJ and significantly hindering DNA repair over time.

## Results

### DNA damage induces a switch in the respective levels of A-family cyclins

**Figure 1:**
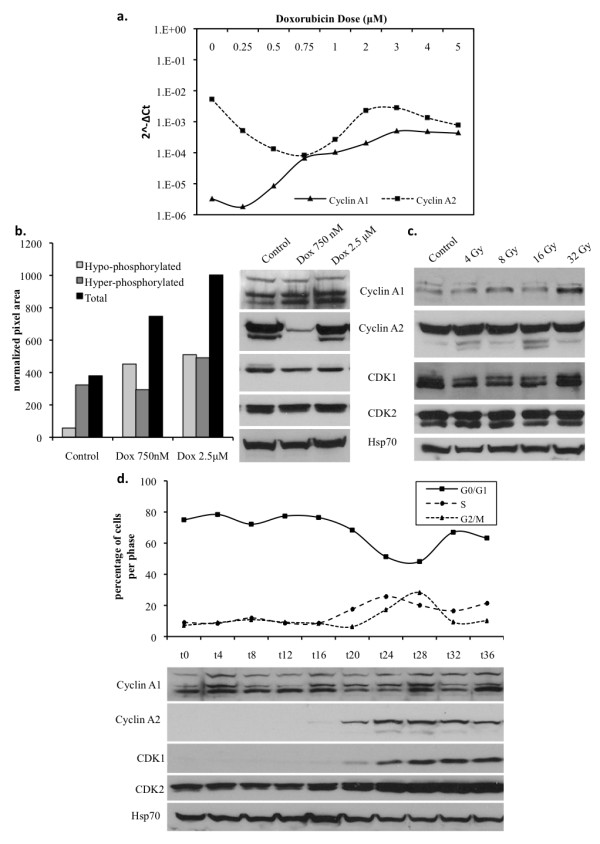
**DNA DSBs induce an upregulation of cyclin A1 but not cyclin A2 in A549 cells in a cell cycle-independent manner A) Relative expression levels respect to GAPDH (2^^-ΔCt^) of cyclin A1 (*CCNA1*) vs. cyclin A2 (*CCNA2*) mRNA after 24 hours of treatment with increasing doses of Doxorubicin (250 nM to 5 μM)**. B) Western blot analysis of cyclin A1, cyclin A2, CDK1 and CDK2 expression levels with Hsp70 as a loading control after 24 hours of treatment with Doxorubicin (Dox 750 nM and 2.5 μM). Quantification of cyclin A1 expression levels as normalized pixel area respect to Hsp70. C)Western blot analysis of protein expression 1 hour after administration of increasing doses of γ-irradiation (4 Gy to 32 Gy). D) Flow cytometry cell cycle analysis with corresponding western blot showing cyclin A1, cyclin A2, CDK1 and CDK2 expression levels over the course of the synchronous cell cycle induced by serum starvation.

We first compared mRNA levels of both members of the cyclin A family after treatment with increasing doses of Doxorubicin (from 250 nM up to 5 μM), a well-known inducer of DNA DSBs. We found that cyclin A1 upregulation is dose dependent with a plateau that is reached around 2.5 μM (IC90). On the contrary, Doxorubicin treatment caused a downregulation of cyclin A2 mRNA levels with a nadir that is reached at the dose of 750 nM (IC50) followed by a relative increase close to basal levels (that are not reached) at a dose of 2.5 μM (IC90) and further followed by a constant decline at higher doses (Figure 1A).

These finding were congruent with protein levels of both cyclins A1 and A2 (Figure 1B). The cyclin A1 antibody we utilized detected two bands, which both augmented upon treatment. The upper band we hypothesized to be a phosphorylated or hyper-phosphorylated form of cyclin A1, which was barely detectable when phosphatase inhibitors were excluded from the lysis buffer. The lower band a hypo-phosphorylated or non-phosphorylated form, which was detectable when cell lysis was performed with or without phosphatase inhibitors (Additional File 1). Relative quantification of bands showed that Doxorubicin, while inducing a slight increase in the hyper-phosphorylated form of cyclin A1, induced a marked dose-dependent increase in the hypo-phosphorylated form. These finding were also noted in A549 cells 1 hour after gamma-irradiation (Figure 1C) suggesting that cyclin A1 upregulation is not specific to doxorubicin treatment and that the timing of its upregulation is compatible with DNA repair events.

To ensure that the increase in cyclin A1 expression observed was not a result of cell cycle redistribution, we analyzed the expression of cyclin A family members during the synchronous cell cycle in the A549 NSCLC cell line. We observed that unlike cyclin A2, which, as expected, was expressed during the S and G2/M phases, cyclin A1 remained fairly constant throughout the cell cycle (Figure 1D). Cell cycle analysis by flow cytometry was also performed on asynchronous A549 cells treated for 24 hours with Doxorubicin (750 nM and 2.5 μM) in comparison to untreated controls, and as expected Doxorubicin treatment resulted in an activation of DNA damage cell cycle checkpoints at G1-S and G2-M phase transitions (Additional File 2). Cells treated with 750 nM Doxorubicin exhibited a decrease in the percentage of cells in S phase, which is duly noted by the observed decrease in cyclin A2 expression levels. However, treatment with 2.5 μM Doxorubicin resulted in a relative increase in the percentage of cells in S phase, which mirrors the increase in cyclin A2 expression at higher doses of Doxorubicin as seen by western blot. These data confirm that cyclin A1 is strongly induced under DNA damaging conditions and also supports a DNA damage-induced molecular switch between cyclin A2 and cyclin A1 during genotoxic stress.

### Cyclin A1 localizes to the nucleus during genotoxic conditions and its overexpression increases in vitro NHEJ activity

**Figure 2:**
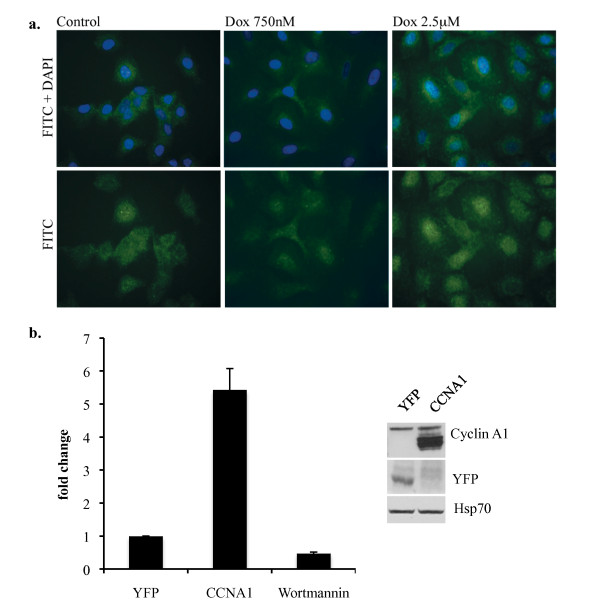
**Nuclearization of cyclin A1 under DNA DSB conditions and its role in NHEJ**. A) Immuno-fluorescence analysis by fluorescent microscopy of cyclin A1 localization in A549 cells after treatment with Doxorubicin (750 nM and 2.5 μM). Lower panels show FITC-stained cyclin A1 expression (green) and upper panels show FITC and DAPI (blue) merge at 400× magnification. B) Fold change, respect to YFP, of *in vitro *NHEJ plasmid re-ligation activity as quantified by real time PCR in HEK293FT cells transiently transfected with *YFP *(control) or cyclin A1 (*CCNA1*) and respective western blot and ponceau S staining verifying overexpression respect to Hsp70.

To determine if cyclin A1 upregulation under DNA damaging conditions was specific to a sub-population or was found in all cells we performed flow cytometry analysis of Doxorubicin treated A549 cells. Cyclin A1 upregulation was observed in all cells, further confirming that this was independent of the cell cycle (data not shown). We also analyzed Doxorubicin treated A549 cells by immunofluorescence staining and microscopy noting not only a dose-dependent increase in fluorescent signal but also a nuclear localization of cyclin A1 protein at higher doses of Doxorubicin (2.5 μM) treatment (Figure 2A). The nuclear localization and the dose-dependent increase in cyclin A1 expression could speak further towards a specific role for cyclin A1 in DNA repair mechanisms.

To address the role of cyclin A1 in DNA DSB repair mechanisms, we used an *in vitro *plasmid re-ligation assay based on the ability of the whole cellular extract to re-join a linearized plasmid. Wortmannin, a known inhibitor of DNA dependent protein kinase (DNA PK), was used as a control to demonstrate the dependency of re-ligation upon NHEJ. Quantification of plasmid re-ligation was performed by real-time PCR utilizing primers, which bound both upstream and downstream of the enzymatic cut site, amplifying only upon re-ligation of plasmid DNA, and values were normalized on the quantity of plasmid in each reaction by primers which bound an intact region of plasmid DNA. We analyzed the NHEJ capability of HEK293FT cells (utilized for their optimal transfection efficiency), transiently transfected to overexpress cyclin A1 or enhanced yellow fluorescent protein (YFP, negative control). In cells overexpressing cyclin A1 there was a significant increase (approximately 6-fold) in NHEJ activity respect to YFP controls (Figure 2B).

### Roscovitine, at doses primarily inhibiting CDK2, but not CDK7 or 9 prevents DNA damage-induced cyclin A1 transcriptional upregulation and increases protein degradation

**Figure 3:**
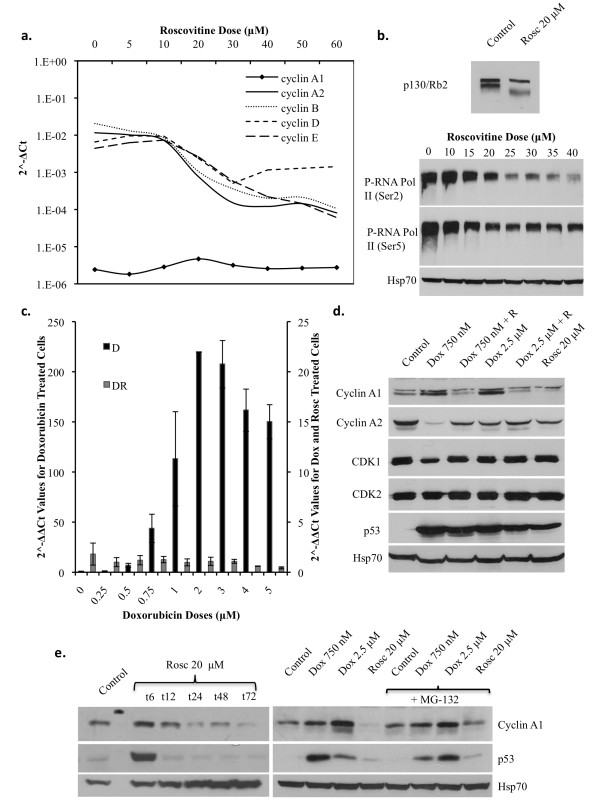
**Roscovitine inhibits DNA DSB-induced upregulation of cyclin A1 mRNA at doses primarily affecting CDK2 and post-translationally downregulates cyclin A1 protein levels over time in A549 cells**. A) Expression levels respect to GAPDH (2^^-ΔCt^), in mRNA of cyclin A1, cyclin A2, cyclin B, cyclin D and cyclin E after 24 hours of treatment with increasing doses of Roscovitine (5-60 μM). B) (Upper blot) Western blot analysis of inhibitory activity of Roscovitine (Rosc) against CKD2 phosphorylation of p130/Rb2 as shown by a shift in p130/Rb2 band height from hyper-phosphorylated in control cells to hypo-phosphorylated in Roscovitine treated cells, upper band is non-specific. (Lower blot) Western blot analysis of Roscovitine inhibition of CDK7 and CDK9 phosphorylation of the C-terminal domain (CTD) of RNA polymerase II, on serine 5 and serine 2 respectively, in cells treated for 24 hours with increasing doses of Roscovitine (10-40 μM). C) Fold change, respect to control (2^^-ΔΔCt^), of cyclin A1 mRNA expression levels in cells treated with either increasing doses of Doxorubicin alone (250 nM to 5 μM) or increasing doses of Doxorubicin in combination with 20 μM Roscovitine for 24 hours. Note that black bars represent Doxorubicin only treated cells and correspond to the vertical axis on the left-hand side of the graph, while grey bars represent Doxorubicin and Roscovitine treated cells and correspond to the vertical axis on the right-hand side of the graph. D) Western blot analysis of cyclin A1, cyclin A2, CDK1 and CDK2 protein expression in cells treated for 24 hours with either Doxorubicin (750 nM or 2.5 μM) alone, 20 μM Roscovitine alone, or in combination (Dox 750 nM/2.5 μM + R). p53 protein expression was included as a control for drug treatments. E) Post-translational inhibition of cyclin A1 protein levels over time. (Left-side blot) cyclin A1 and p53 protein expression in cells treated for increasing amounts of time (6-72 hours) with 20 μM Roscovitine. (Right-side blot) cyclin A1 and p53 expression in cells treated for 24 hours with either Doxorubicin (750 nM and 2.5 μM) or 20 μM Roscovitine alone or in combination with 10 μM of the proteosome inhibitor MG-132.

Roscovitine, being a CDK2 inhibitor, can depress E2F-dependent transcription by blocking the phosphorylation of Rb-family proteins. Cyclin A1 expression is not E2F-dependent[30], therefore we investigated the effects of Roscovitine on cyclin A1 basal expression and eventually on the DNA damage-induced upregulation. First we analyzed the mRNA expression levels of cyclins A1, A2, B, D, and E after 24 hours of incubation with increasing doses (up to 60 μM) of Roscovitine. We found that all cyclin mRNA expression levels were greatly reduced respect to untreated controls (Figure 3A), except for cyclin A1, whose basal levels were substantially lower than the other cyclins and were not downregulated but remained fairly constant upon Roscovitine treatment consistent with its E2F-independent transcriptional regulation (Figure 3A). Therefore, we treated A549 cells for 24 hours with increasing doses of Doxorubicin (as previously stated) alone or in combination with a fixed dose of 20 μM Roscovitine. We chose to use the dose of 20 μM as it is not only a dose commonly utilized in the literature but also as it was experimentally proven to preferentially inhibit CDK2 resulting in a hypo-phosphorylation of p130/Rb2, while it is the highest dose with a limited effect on CDK7 and CDK9, as shown by the phosphorylation of the C-terminal domain (CTD) of RNA Polymerase II on serine 5 and 2 respectively (Figure 3B). Roscovitine was able to completely abolish the Doxorubicin-induced cyclin A1 mRNA and protein upregulation (Figure 3C&3D) suggesting that a residual CDK2 activity is required for cyclin A1 upregulation. Furthermore, co-administration of Doxorubicin and Roscovitine resulted in a change in cyclins A2, B, D and E mRNA expression levels, respect to Doxorubicin treatment alone (Additional File 3). In particular, cyclin A2 mRNA levels demonstrated an attenuated variation during combination treatments, which is consistent with the cell cycle distribution as observed by flow cytometry (Additional File 2). At the protein level, the combination of Roscovitine with Doxorubicin resulted in an inversion of the Doxorubicin-induced molecular switch between cyclin A1 and cyclin A2 (Figure 3D).

Unlike cyclin A1 mRNA levels, treatment with Roscovitine alone also resulted in a decrease in cyclin A1 protein expression over time (Figure 3D&3E), suggesting that, aside from transcriptional regulation, Roscovitine may also regulate cyclin A1 on a post-transcriptional level. To confirm this hypothesis we treated A549 cells with Doxorubicin and Roscovitine respectively as well as 10 μM of the proteosome inhibitor MG-132. Inclusion of MG-132 significantly prevented the downregulation of cyclin A1 protein levels after treatment with 20 μM Roscovitine (Figure 3E). The transcriptional and post-transcriptional regulation of cyclin A1 by Roscovitine was confirmed in a panel of NSCLC (A549 and H23), breast (MCF-7 and MDA-MB-231) and prostate cancer (LNCAP and DU145) cell lines (data not shown).

### Combined treatment with Roscovitine and Doxorubicin results in a downregulation of NHEJ capability

**Figure 4:**
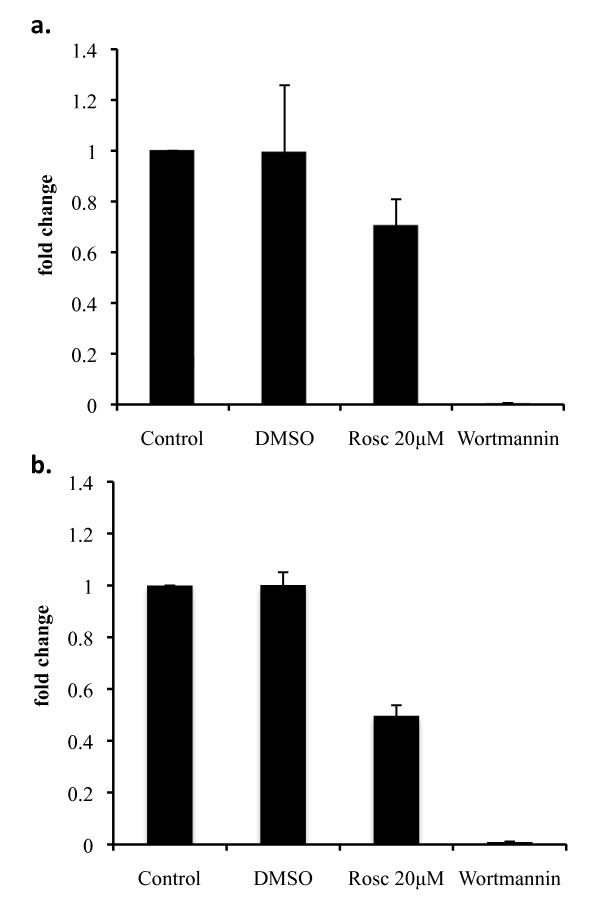
**Roscovitine inhibits NHEJ activity synergistically when combined with Doxorubicin treatment in A549 cells**. A) Analysis by real time PCR of NHEJ plasmid re-ligation activity of untreated A549 cell lysate with the addition of 20 μM Roscovitine, DMSO or Wortmannin. B) Analysis by real time PCR of NHEJ plasmid re-ligation activity in A549 cells treated for 12 hours with 20 μM Roscovitine. Wortmannin was added to untreated cell lysate as a negative control for NHEJ activity *in vitro*.

Cyclin A1 knockout MEFs have shown a reduced NHEJ capability[27]. To determine if Roscovitine may have a comparable effect on NHEJ mechanisms, we incubated untreated A549 cell lysates with 20 μM Roscovitine, DMSO, or Wortmannin for 15 minutes prior to incubation with linearized plasmid. While Wortmannin was able to almost completely inhibit NHEJ activity, DMSO had no effect and Roscovitine resulted in an approximate 25% diminution in plasmid re-ligation, which can be accounted for by a direct inhibition of CDK activity and eventual off-target effects of the drug (Figure 4A). However, when lysates from A549 cells treated for 12 hours with 20 μM Roscovitine were assayed for NHEJ capability, they demonstrated an approximate 45% reduction in plasmid re-ligation (Figure 4B) as a result of an additional biological mechanism to the pharmacological inhibition of CDK2.

### Roscovitine enhances Doxorubicin-induced DSBs and delays DNA damage repair over time

**Figure 5:**
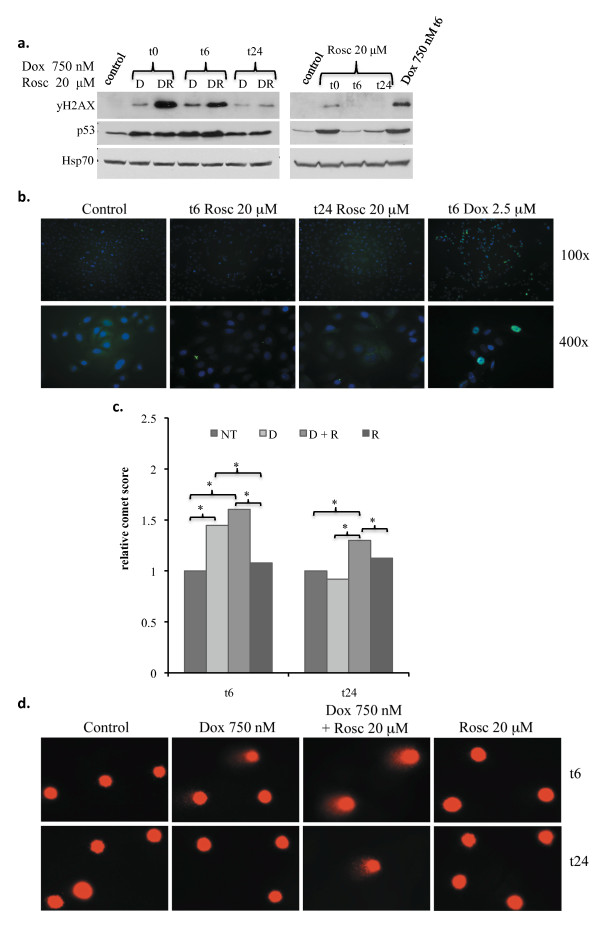
**Roscovitine when combined with Doxorubicin increases DNA DSBs and overall DNA damage over time in A549 cells**. A) Western blot analysis of DNA DSBs by phosphorylated γH2AX (serine 139) immediately (t0) or 6 (t6) and 24 (t24) hours following a 6 hour treatment with either 750 nM Doxorubicin (D) or 20 μM Roscovitine alone or in combination (DR). B) Immunofluorescence analysis by fluorescent microscopy of phosphorylated γH2AX (serine 139) at the abovementioned time points following 6 hours of treatment with 20 μM Roscovitine or 2.5 μM Doxorubicin (as a positive control for DSBs). Images shown are γH2AX (FITC) and DAPI merges under 100× (upper panels) and 400× (lower panels) magnifications. C) Alkaline comet assay quantification and D) respective images (400x magnification), 6 (t6) and 24 (t24) hours following a 6 hour incubation with abovementioned treatments (Control, NT; Doxorubicin, D; Doxorubicin + Roscovitine, D+R; Roscovitine, R) to measure overall DNA damage.

To determine if the inhibition of NHEJ activity led to an overall increase in DNA DSBs we analyzed the quantity of phosphorylated γH2AX by western blot (Figure 5A). After six hours of incubation with respective drug treatments, we removed the drug-containing medium and analyzed A549 cells for γH2AX phosphorylation immediately following the six hour treatment(t0), then six(t6) and 24(t24) hours after drug removal with respect to control cells. Doxorubicin treatment induced an activation of γH2AX, which was significantly augmented following combined treatment with Roscovitine over time (Figure 5A), even though Roscovitine alone did not significantly activate γH2AX as shown by western blot and immunofluorescence staining (Figure 5A&5B).

In addition to γH2AX, we observed overall DNA damage on a single-cell level utilizing the alkaline comet assay. The comet assay revealed no significant differences in DNA damage between cells treated with only Doxorubicin and those treated with both Doxorubicin and Roscovitine six hours-post drug removal. However, 24 hours after drug removal, while Doxorubicin-only treated cells had completely repaired the damage, cells treated with both Doxorubicin and Roscovitine contained a greater amount of DNA damage (p ≤ 0.0001) (Figure 5C&5D). These data further support the hypothesis that Roscovitine can augment Doxorubicin-induced DNA damage by hindering DSB repair over time.

### Combined treatment leads to global changes in DNA repair pathways

**Figure 6:**
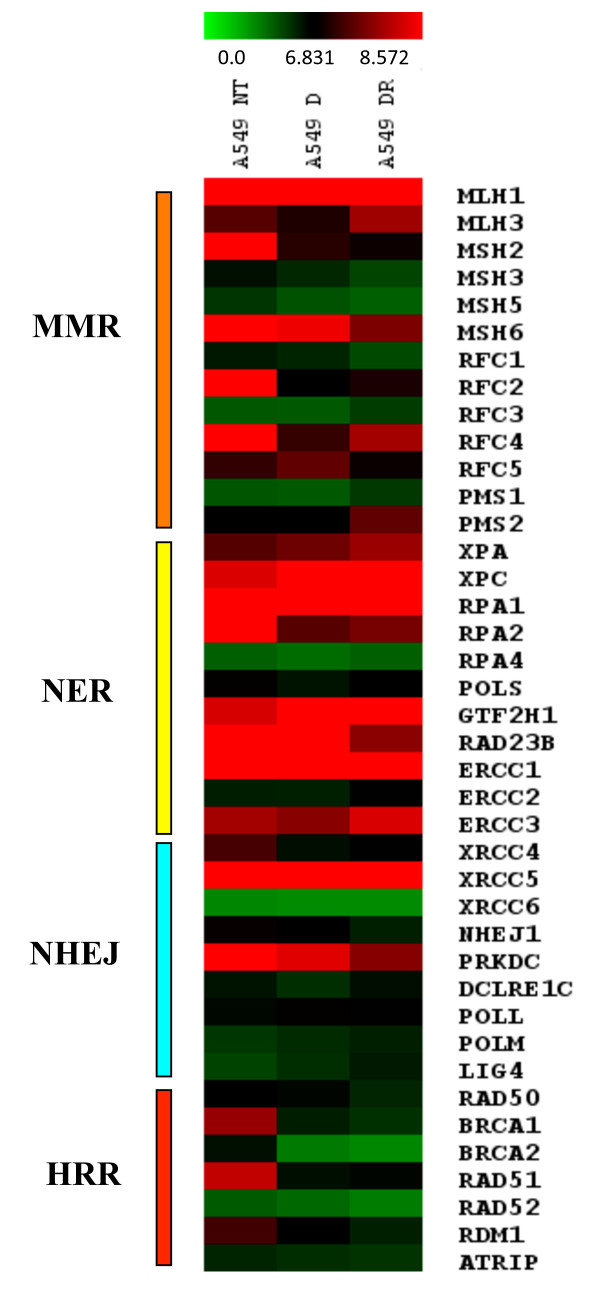
**Combination treatment with Roscovitine globally affects DNA repair pathways**. Corrected microarray signal values of genes involved in DNA repair clustered by specific DNA repair pathway of A549 cells treated for 24 hours with 1 μM Doxorubicin alone or in combination with 20 μM Roscovitine in comparison to control cells.

To assess the global effects of combination treatment, we performed genome-wide microarray analysis on cRNA from A549 cells treated for 24 hours with either 1 μM Doxorubicin alone or in combination with 20 μM Roscovitine. Here we focus our analysis primarily on genes involved in the DNA repair pathways: mismatch repair (MMR), nucleotide excision repair (NER), homologous recombination (HR), and NHEJ. We grouped the genes related to these pathways that changed in a statistically significant manner (p-value ≤ 0.05) after combination treatment respect to Doxorubicin treatment in Table 1 and Figure 6. The most significant changes were observed in the NHEJ and HR pathways. In particular in HR we observed a decrease in *BRCA1 *(fold change: -0.46) and *RAD50 *(-0.75). Furthermore, there were significant variations in key genes involved in NHEJ. In particular, we observed a significant decrease in the expression levels of Ku80 (*XRCC5 *-0.61), DNA-activated protein kinase (*PRKDC *-0.61), and NHEJ1 (-0.80) (Table 1 and Figure 6). These data support the reduced NHEJ activity observed with the *in vitro *NHEJ plasmid re-ligation assay. Moreover, they demonstrate a more global affect on DNA repair pathways as a result of combination treatment with Roscovitine.

**Table 1:** Statistically significant genes involved in DDR after combination treatment

ID AFFYMETRIX	Gene symbol	A549 D1	A549 D2	A549 DR1	A549 DR2	M	P.Value
**Signal**							

223598_at	*RAD23B*	8.83	8.91	7.68	7.88	-1.09	0.000223

202996_at	*POLD4*	10.01	10.14	8.89	9.29	-0.98	0.001349

209084_s_at	*RFC1*	5.67	5.77	4.87	4.76	-0.90	0.000436

219418_at	*NHEJ1*	6.76	6.55	5.75	5.96	-0.80	0.001689

211450_s_at	*MSH6*	8.46	8.47	7.61	7.76	-0.78	0.001138

209349_at	*RAD50*	6.40	6.48	5.63	5.75	-0.75	0.001394

203720_s_at	*ERCC1*	9.57	9.65	8.78	8.98	-0.73	0.002189

205887_x_at	*MSH3*	5.71	5.56	5.03	4.85	-0.69	0.003738

219715_s_at	*TDP1*	7.94	7.81	7.26	7.12	-0.68	0.002669

210543_s_at	*PRKDC*	8.36	8.36	7.78	7.72	-0.61	0.00473

208643_s_at	*XRCC5(Ku80*)	9.94	10.06	9.31	9.46	-0.61	0.00434

213734_at	*RFC5*	7.64	7.37	6.91	7.03	-0.53	0.014248

212525_s_at	*H2AFX*	6.05	6.17	5.51	5.69	-0.51	0.011937

211851_x_at	*BRCA1*	5.84	5.93	5.39	5.46	-0.46	0.022329

204752_x_at	*PARP2*	7.89	7.95	7.50	7.65	-0.34	0.049

205672_at	*XPA*	7.63	7.54	7.89	7.87	0.29	0.03678

221143_at	*RPA4*	3.79	4.06	4.25	4.26	0.33	0.01878

1053_at	*RFC2*	6.83	6.61	7.05	7.07	0.34	0.049

227766_at	*LIG4*	5.56	5.40	6.11	5.88	0.52	0.025825

202176_at	*ERCC3*	7.84	7.70	8.31	8.30	0.54	0.006878

209903_s_at	*ATR*	8.11	7.93	8.64	8.53	0.57	0.009919

202451_at	*GTF2H1*	8.60	8.55	9.29	9.07	0.61	0.01218

232134_at	*POLS*	6.32	6.00	6.98	6.75	0.71	0.008367

231119_at	*RFC3*	4.31	4.56	4.95	5.35	0.72	0.008497

204023_at	*RFC4*	7.26	7.17	8.04	7.84	0.72	0.00282

222233_s_at	*DCLRE1C*	5.50	5.44	6.41	6.10	0.78	0.00239

213468_at	*ERCC2*	5.82	5.85	6.58	6.64	0.78	0.000828

209805_at	*PMS2*	6.67	6.74	7.56	7.43	0.79	0.000908

209805_at	*PMS2*	6.67	6.74	7.56	7.43	0.79	0.000908

1554743_x_at	*PMS1*	4.32	4.51	5.29	5.16	0.81	0.002444

204838_s_at	*MLH3*	7.13	7.05	7.97	7.86	0.83	0.001711

Genes involved in DNA repair mechanisms, those shown either decreased or increased in expression level (p value ≤ 0.05) after combination treatment with 1 μM Doxorubicin and 20 μM Roscovitine as compared to 1 μM Doxorubicin only, in A549 cells after 24 hours of treatment.

## Discussion

Under genotoxic conditions the CDK2/cyclin A1 complex increases its functional kinase activity and the ability to phosphorylate Ku70. In addition, here we demonstrated upon treatment with different DNA damaging agents (doxorubicin or γ-irradiation) a marked dose dependent increase in the RNA and protein levels of cyclin A1, which is independent of cell cycle phase redistribution. Conversely cyclin A2 (whose expression is tightly related to the S and G2-M phases of the cell cycle) is downregulated under genotoxic stress conditions as a result of the check-point activation and consequent decrease of the S phase fraction. This switch in the respective levels of the A-family cyclins may be functionally relevant to redirect CDK2 activity toward DNA repair, especially given the findings that the ectopic overexpression of cyclin A1 increased *in-vitro *NHEJ activity and that cyclin A1 depletion, as demonstrated by others[27], results in an impaired DNA DSB repair ability.

DNA DSBs are considered the most lethal form of DNA damage and CDK inhibition has been shown to potentially affect the two major DSB repair pathways (HR and NHEJ)[7]. Various mechanisms have been proposed to explain this effect such as the deregulation of the DNA damage-induced checkpoint signalling cascade[13] or the downregulation of specific genes involved[33,34]. Roscovitine is an oral 2,6,9 trisubstituted purine analog currently under phase II investigation, which competes with ATP for the catalytic binding site on CDK2 (but also CDKs 1, 7 and 9 with a much lower affinity) with a demonstrated antitumor activity in many human cancer models and a nice toxicity profile.

One of the most prominent effects of the drug is the inhibition of CDK2/cyclin E complexes, which causes a decrease in Rb phosphorylation and a consequent inactivation of E2F family members, thus leading to cyclin transcriptional downregulation and ultimately to cell cycle arrest. This strong transcriptional depression of most of the cell cycle related cyclins further enforces the drug's inhibitory effect on CDK/cyclin complexes. Furthermore, Roscovitine has been shown to downregulate several other genes involved in a wide spectrum of cellular functions[35,36], probably as a result of partial CDK7/cyclin H and CDK9/cyclin T inhibition[37]. In addition, whole genome ChIP-on-chip analysis recently mapped E2F transcription factor family members to the promoters of many more genes than were traditionally associated with the cell cycle[38], suggesting an alternative mechanism to explain these transcriptional effects.

We investigated the effects that Roscovitine may have on cyclin A1 transcription as one of the possible mechanisms through which CDK2 inhibition may curb DNA DSB repair activity. The promoter of the cyclin A1 gene, *CCNA1 *is not E2F-dependent and, consistently, increasing doses of Roscovitine did not repress cyclin A1 basal transcription levels in contrast to cyclins A2, B, D and E. However, we demonstrated that Roscovitine at doses preferentially inhibiting CDK2 but not CDK7 and 9 completely abolished cyclin A1 DNA damage-induced upregulation, thus suggesting that residual CDK2 activity is required for cyclin A1 upregulation. In addition Roscovitine co-administered with doxorubicin was able to largely modify the patterns of cell cycle phase distribution in comparison to doxorubicin only treatment. This resulted in an augmented S phase and consequently in an increased expression of cyclin A2. The combined treatment thus resulted in the complete inversion of the doxorubicin-induced switch between cyclin A1 and cyclin A2.

Roscovitine, alone or under DNA damaging conditions, was able to diminish cyclin A1 protein levels as well. Such transcriptional and post-transcriptional repression was observed in different NSCLC, prostate and breast cancer cell lines and we propose that this potentiates and synergizes the Roscovitine-mediated CDK2 inhibition thus resulting in a significant decrease of cellular NHEJ ability. In fact, we observed that combination treatment led to an increase in DNA DSBs and overall DNA damage over-time, further substantiating, not only the importance of CDK-inhibitors in combination therapy but also the role of CDKs in DNA repair mechanisms. While these findings were supported by genome-wide mircroarray analysis, we also observed a significant effect on key genes involved in other DNA repair pathways.

## Conclusions

Roscovitine has shown to be able to significantly modify the DDR response. Even considering the many genes that are potentially involved, the putative role of CDK2 in multiple DDR pathways along with the downregulation of cyclin A1, may further explain the effective inhibition of a broad range of DNA repair mechanisms by Roscovitine. In particular since NHEJ is considered the major pathway for the repair of γIR-induced DNA DSBs in human cells[39], we believe our data support further investigation on the therapeutic advantages of combination therapy with Roscovitine and Radiotherapy.

## Methods

### Cell Culture and Serum Starvation

The following solid cancer human cell lines were purchased from and authenticated by American Type Culture Collection (ATCC; Manassas, VA) and cultured at 37°C in a humidified atmosphere of 5% CO_2 _in air, within the appropriate medium according to supplier recommendations supplemented with 10% (v/v) heat-inactivated fetal bovine serum (Atlanta Biologicals; Lawrenceville, GA) and 100U of Penicillin and 100 μg/ml of Streptomycin (Sigma-Aldrich; St. Louis, MO): NSCLC cell lines A549 and H23, breast cancer cell lines MCF-7 and MDA-MB-231, prostate cancer cell lines LNCAP and DU145, and the adenovirus transformed human embryonic kidney epithelial cells HEK293FT. Cells were regularly sub-cultured according to ATCC recommendations with a 0.25% trypsin-EDTA solution (Sigma). To obtain synchronous populations of cells, confluent plates of A549 cells were incubated in media supplemented with 0.1% (v/v) heat-inactivated fetal bovine serum for 96 hours. Cells were then sub-cultured into serum-containing medium and time points were taken every four hours.

### Drugs, irradiations and treatments

Doxorubicin was obtained from BioMol International (Plymouth Meeting, PA). Lyopholized drug was re-suspended into a 1:1 mixture of dimethyl sulfoxide (DMSO; Fisher Scientific; Pittsburgh, PA) and MilliQ filtered H_2_O (Millipore; Bellerica, MA) to a concentration of 4.31 mM, aliquoted for use and stored at -20°C. Roscovitine was obtained from Signa Gen Laboratories (Gaithersburg, MD). Lyophilized drug was re-suspended into DMSO to a concentration of 14.1 mM, aliquoted and stored at -20°C until use. Fresh dilutions from the stock solutions were prepared for each treatment. Taxol was obtained from USB Corporation (Cleveland, OH). Lyophilized drug was re-suspended into DMSO to a concentration of 5.86 mM, aliquoted and stored at -20°C until use. MG-132 (Z-Leu-Leu-Leu-al) was obtained from Sigma. Lyophilized drug was re-suspended into DMSO to a concentration of 10 mg/ml, aliquoted and stored at -20°C until use. Irradiations were performed in an AECL Gamma Cell 40, Cs-137 irradiator at a dose rate of 1 Gy/minute for respective doses. In treatments throughout this article the control samples refer to cells treated with an equal concentration (v/v) of DMSO as in the highest drug concentration used per experiment.

### Western Blot Analysis and SDS-PAGE

Equal amounts (50-100 μg) of whole cell lysates were resolved by SDS-PAGE and transferred to a nitrocellulose membrane (Whatman Inc., Piscataway, NJ) by wet electrophoretic transfer. Non-specific binding sites were blocked for 1 hour at room temperature with 3% non fat dry milk (NFM) in tris-buffered saline containing 0.01% Tween-20 (TBS-T) and probed with the following primary antibodies in 3% NFM in TBS-T overnight at 4°C; rabbit anti-cyclin A1 (sc-15383; Santa Cruz Biotechnology Inc.; Santa Cruz, CA), mouse anti-cyclin A2 (CY-A1; Sigma), mouse anti-cdc2 (A17; Abcam, Cambridge, MA), rabbit anti-CDK2 (sc-163; Santa Cruz), rabbit anti-p53 (sc-6243; Santa Cruz), mouse anti-Hsp70 (sc-24; Santa Cruz), mouse anti-p130/Rb2 full length (610262; BD Biosciences, San Jose, CA), rabbit anti-serine 952 phosphorylated p130/Rb2 (sc-16298; Santa Cruz), rabbit anti-serine-2 phosphorylated RNA polymerase II (A300-654A; Bethyl Laboratories Inc., Montgomery, TX), rabbit anti-serine-5 phosphorylated RNA polymerase II (A300-655A; Bethyl), mouse anti-α-tubulin (sc-58666; Santa Cruz), and mouse anti-ser139 phosphorylated histone γH2AX (Millipore cat. #05636; lot# DAM1567248). Membranes were washed for 15 minutes in TBS-T and then incubated for 1 hour with either goat anti-mouse (31432; Pierce; Rockford, IL) or mouse anti-rabbit (31464; Pierce) horseradish peroxidase conjugated IgG at a dilution of 1:10,000 in 3% NFM in TBS-T. This was followed by 15 minutes of wash in TBS-T and enhanced chemiluminescence (ECL; Amersham, Buckinghamshire, UK) according to the manufacturer's instructions. All western blot images included in article are representative of at least three consecutive independent experiments.

### Immunostaining

Following respective drug treatments, cells grown directly on sterilized glass coverslips were fixed and permeabilized for 10 minutes in 70% cold methanol (MeOH), immunostained (for cyclin A1 and γH2AX) and analyzed as previously described[40].

### Flow cytometry

Cells (1 × 10^6^) were collected, after respective drug treatments, washed, resuspended in 1 ml of PBS and fixed and permeabilized for at least 10 minutes in 70% cold ethanol. After fixation cells were pelleted, washed 3 times with PBS, re-suspended into a primary antibody solution (10 μg/ml antibody diluted in PBS) and incubated on ice for 15 minutes. Cells were then pelleted, washed 3 times with PBS, re-suspended into FITC-conjugated secondary antibody solution (10 μg/ml) and incubated for 15 minutes on ice protected from the light. Cells were washed 3 times in PBS and re-suspended in propidium iodide staining solution, 10 μg/ml propidium iodide (from stock of 0.5 mg/ml in 0.38 mM sodium citrate pH 7.0) and 25 μg/ml DNase-free RNase A (from stock of 10 mg/ml RNase A in 10 mM Tris pH 7.5 and 15 mM NaCl) diluted in PBS. Cells were incubated at 37°C for a minimum of 30 minutes protected from light and analyzed immediately by flow cytometry utilizing an Epics XL-MCL BeckmanCoulter (The Wistar Institute, Philadelphia, PA). Graphs represent average fluorescence intensity or average percentage of cells found in cell cycle phase over three consecutive independent experiments.

### Reverse Transcriptase-PCR and Real time (RT-PCR)

Total RNA from cell lines was extracted using the High Pure RNA Isolation Kit (Roche) following the manufacturer's instruction. cDNA was synthesized from 1 μg of total RNA by using random hexamers as primers and moloney murine leukemia virus reverse transcriptase (Invitrogen, Carlsbad, CA) according the manufacturer's protocol in a final volume of 20 μl. As a control for genomic contamination a reverse transcription (RT) reaction was carried out without the addition of the reverse transcriptase (RT-). After cDNA synthesis, samples were diluted 1:10 and 4 μl was used in each real time polymerase chain reaction (real time PCR). cDNA was amplified using species specific intragenic primers for *CCNA1*[23], *CCNA2*, *CCNB1*, *CCND3*, *CCNE1*, *TP53 *and *GAPDH *genes (Additional File 4). Real time PCR was carried out utilizing SybrGreen Master Mix (Roche, Basel, Switzerland) following the manufacturer's instructions in a final reaction volume of 10 μl. Reactions were performed on a LightCycler 480 II (Roche Diagnostics, Indianapolis, IN) with an initial denaturation of 5 minutes at 95°C; 45 cycles of 10 seconds at 95°C, 20 seconds at 60°C, and 10 seconds at 72°C where fluorescence was acquired. Each sample was run in triplicate and data was analyzed using the comparative Ct method with GAPDH as the endogenous control and control cells as the reference sample in each experiment. Final data points represent the average fold change respect to control (2^^-ΔΔCt^) or expression levels respect to GAPDH (2^^-ΔCt^) of at least three consecutive independent experiments.

### Alkaline Comet Assay

After appropriate drug treatments, cells were harvested and analyzed utilizing the alkaline comet assay as previously described[41,42]. Briefly, cells were mixed in a suspension of low melting point agarose and spread on agarose-coated slides. Once the agarose solidified, slides were incubated in lysis buffer followed by electrophoresis to allow migration of DNA and detection of DNA damage. Cells were then stained with 1 μg/mL ethidium bromide and analyzed using the fluorescence microscope Olympus BX40 (Melville, NY) with a Spot-RT digital camera and software (Webster, NY). At least 200 cells were evaluated per experimental point. Visual scoring of comet images using fluorescence microscopy was performed according to Norbury[43]. Briefly, each nucleus is assigned a score from 0-4 depending on the relative intensity of DNA fluorescence in the tail (0 = no damage, 4 = >80% of DNA found in the tail) and the final score is calculated as the average DNA damage found in all cells counted from three consecutive independent experiments. Statistical analysis was carried out using a standard student's t test.

### Transient transfections

The human cyclin A1 IMAGE clone 5172478 (GenBank:BC036346.1) was purchased from ATCC (MGC-34627) transformed into DH5α heat-shock competent *E. coli *cells and grown on LB agar plates or in broth with 100 μg/ml Ampicillin (Fisher) at 37°C. Plasmid DNA was extracted using the Genopure Plasmid Midi Kit (Roche) following manufacturer's instructions then verified by restriction enzyme digestion and gel electrophoresis. HEK293FT cells were transiently transfected using a 6:2 ratio of Fugene HD (Roche) and plasmid DNA (2 μg) following manufacturer's protocol. Enhanced yellow fluorescent protein (pEYFP) plasmid DNA was utilized as a control for transfection efficiency at the same concentration. Cells were analyzed after 36 hours of transfection by western blot and fluorescence microscopy to confirm expression of transfected protein and then utilized in experiments as described.

### In vitro NHEJ assay

The *in vitro *NHEJ assay was performed on respectively treated cell lysates as previously described[44] utilizing 120 μg of protein extract and 60 μg of purified BamHI (Roche) digested pCI-neo plasmid DNA (Promega). A reaction including the incubation of 20 μM Wortmannin with whole cellular lysate for 15 minutes on ice before the addition of digested plasmid DNA was included as a negative control for NHEJ activity in each experiment. After incubation samples were diluted 1:10, phenol chloroform 25:24:1 (Fisher) extracted, and ethanol precipitated overnight at 4°C. DNA was resuspended into 20 μl of Tris-EDTA buffer and 1 μl was utilized in each real time PCR reaction. To detect plasmid re-ligation one set of primers amplified an intact region of the plasmid to act as the endogenous control, while a second set of primers bound both up-stream and down-stream of the enzymatic cut site. Samples were run in triplicate with each primer pair following the real-time PCR protocol described above. Final results represent the average fold change (2^^-ΔΔCt^) in re-ligation respect to control, over three consecutive independent experiments.

### Microarray Analysis

Total RNA was isolated by Trizol (Invitrogen). Fifteen μg of total RNA was converted to cDNA by using Superscripts reverse transcriptase (Invitrogen), and T7-oligo-d(T)24 (Geneset) as a primer. Second-strand synthesis was performed using T4 DNA polymerase and E.Coli DNA ligase and them blunt ended by T4 polynucleotide kinase. cDNA was purified by phenol-chloroform extraction using phase lock gels (Brinkmann). Then cDNAs were *in vitro *transcribed for 16 hours at 37°C by using the IVT Labelling Kit (Affymetrix) to produce biotinylated cRNA. Labelled cRNA was isolated by using the RNeasy Mini Kit column (QIAGEN). Purified cRNA was fragmented to 200-300 mer using a fragmentation buffer. The quality of total RNA, cDNA synthesis, cRNA amplification and cRNA fragmentation was monitored by capillary electrophoresis (Bioanalizer 2100, Agilent Technologies). Fifteen micrograms of fragmented cRNA was hybridised for 16 hours at 45°C with constant rotation, using a human oligonucleotide array U133 Plus 2.0 (Genechip, Affymetrix, Santa Clara, CA). After hybridisation, chips were processed by using the Affymetrix GeneChip Fluidic Station 450 (protocol EukGE-WS2v5_450). Staining was made with streptavidin-conjugated phycoerythrin (SAPE)(Molecular Probes), followed by amplification with a biotinylated anti-streptavidin antibody (Vector Laboratories), and by a second round of SAPE. Chips were scanned using a GeneChip Scanner 3000 G7 (Affymetrix) enabled for High-Resolution Scanning. Images were extracted with the GeneChip Operating Software (Affymetrix GCOS v1.4). Quality control of microarray chips was performed using the AffyQCReport software[45]. A comparable quality between microarrays was demanded for all microarrays within each experiment.

### Microarray Statistical Analysis

The background subtraction and normalization of probe set intensities was performed using the method of Robust Multiarray Analysis (RMA) described by Irizarry et al.[46]. To identify differentially expressed genes, gene expression intensity was compared using a moderated t test and a Bayes smoothing approach developed for a low number of replicates[47]. To correct for the effect of multiple testing, the false discovery rate, was estimated from p-values derived from the moderated t test statistics[48]. The analysis was performed using the affylmGUI Graphical User Interface for the limma microarray package[49].

## Abbreviations Used

CDK: cyclin-dependent kinase; DDR: DNA damage response; NHEJ: non-homologous end-joining; DSB: double strand break; HR: homologous recombination; NER: nucleotide excision repair; MMR: mismatch repair.

## Competing interests

The authors declare that they have no competing interests.

## Authors' contributions

MF and CES designed experiments, performed the research, analyzed the data and wrote the paper. DF performed microarray experiments and analysis. FR performed experiments and analyzed the data. LB, AR and AG designed experiments and wrote the paper. All authors critically reviewed and edited the paper.

## Supplementary Material

Additional file 1 **Western blot analysis of cyclin A1 protein expression with and without the inclusion of phosphatase inhibitors in lysis**. Phosphatase inhibitor activity was confirmed by probing for phosphorylated p130/Rb2 in comparison to full-length p130/Rb2. After 24 hours of Doxorubicin treatment (750 nM and 2.5 μM), cyclin A1 protein levels clearly augment in cells lysed with the inclusion of phosphatase inhibitors, whereas the increase is not as notable in cells lysed without the inclusion of phosphatase inhibitors. Click here for file

Additional file 2 **Flow cytometry analysis of cell cycle breakdown after treatment**. Flow cytometry analysis of cell cycle breakdown in A549 cells treated for 24 hours with respective treatments of Doxorubicin (750 nM or 2.5 μM) or 20 μM Roscovitine alone or in combination and graph representing average cell cycle distributions from three consecutive independent experiments. Click here for file

Additional file 3 **Drug induced changed in cyclin mRNA expression levels**. Expression levels respect to GAPDH (2^^-ΔCt^), in mRNA of cyclin A1, cyclin A2, cyclin B, cyclin D and cyclin E after 24 hours of treatment with either increasing doses of Doxorubicin (250 nM to 5 μM) alone or in combination with 20 μM Roscovitine. Click here for file

Additional file 4 **Table of gene specific primer sequences utilized in this manuscript**. Click here for file
