# Supplementary figures and images for: R-Roscovitine (Seliciclib) prevents DNA damage-induced cyclin A1 upregulation and hinders non-homologous end-joining (NHEJ) DNA repair
Source: Mol Cancer. 2010 Aug 4;9:208. doi: 10.1186/1476-4598-9-208 (PMC3224749; doi:10.1186/1476-4598-9-208)

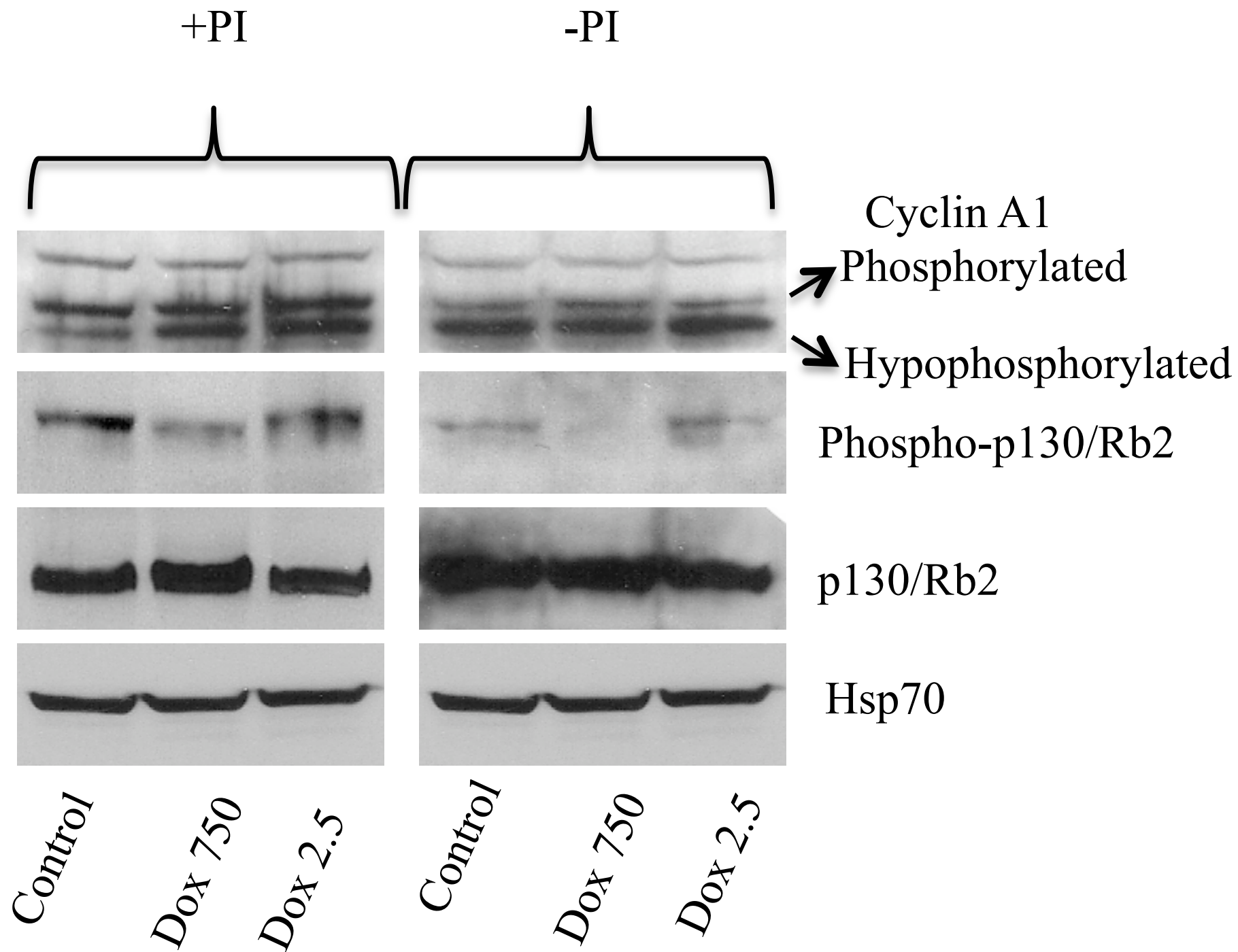

Supplement: Additional file 1 — Western blot analysis of cyclin A1 protein expression with and without the inclusion of phosphatase inhibitors in lysis. Phosphatase inhibitor activity was confirmed by probing for phosphorylated p130/Rb2 in comparison to full-length p130/Rb2. After 24 hours of Doxorubicin treatment (750 nM and 2.5 μM), cyclin A1 protein levels clearly augment in cells lysed with the inclusion of phosphatase inhibitors, whereas the increase is not as notable in cells lysed without the inclusion of phosphatase inhibitors. [file 1476-4598-9-208-S1.PDF]

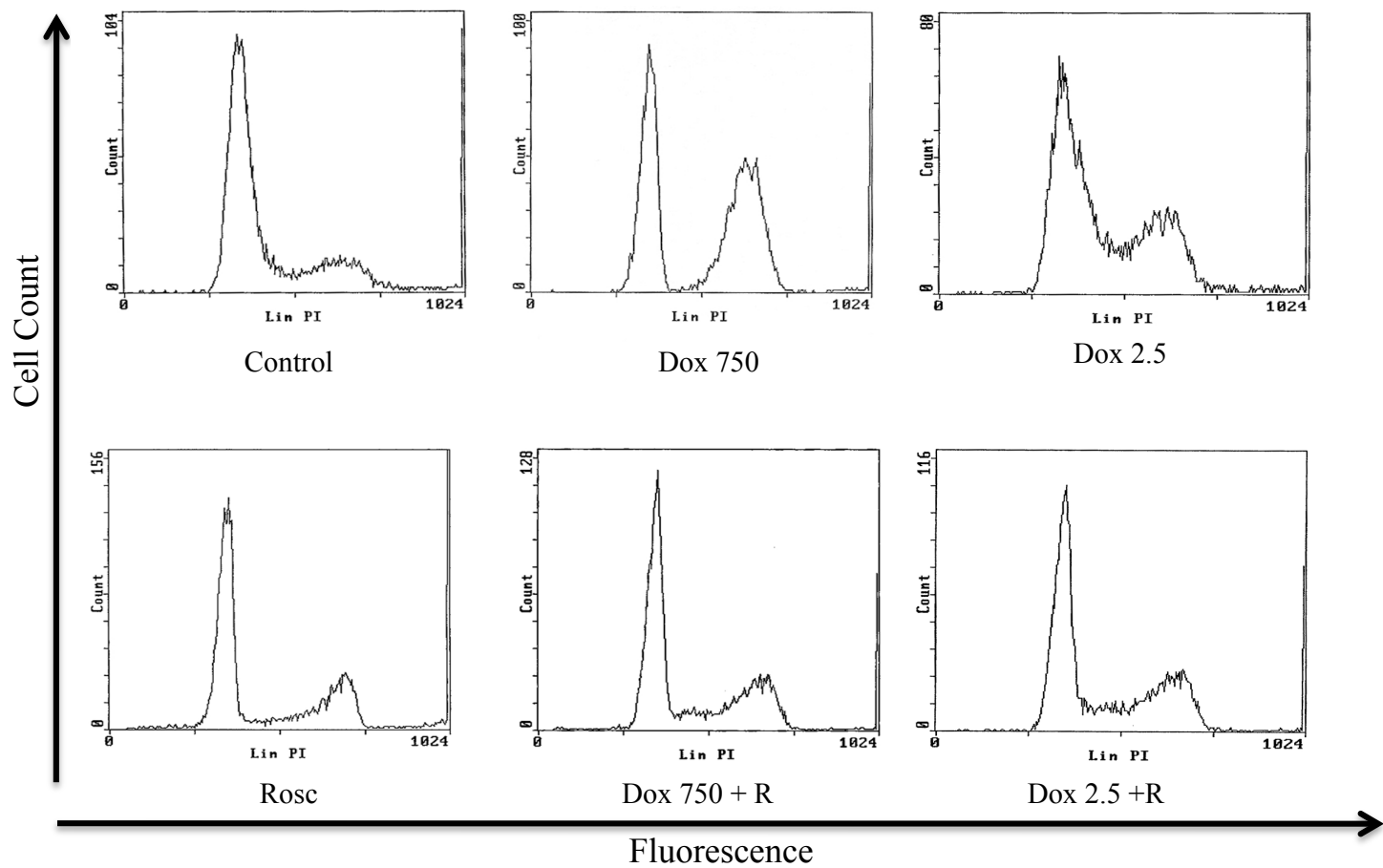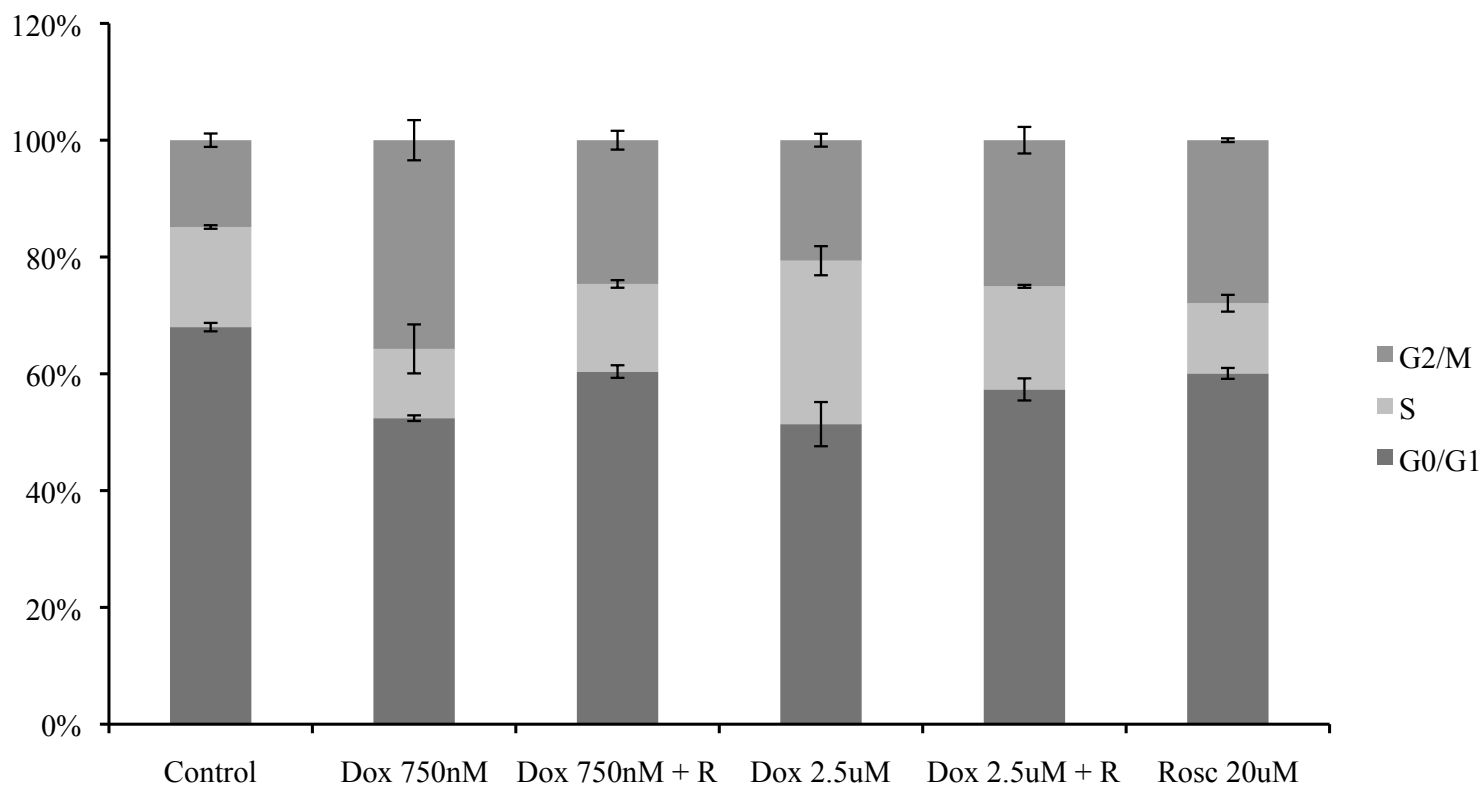

Supplement: Additional file 2 — Flow cytometry analysis of cell cycle breakdown after treatment. Flow cytometry analysis of cell cycle breakdown in A549 cells treated for 24 hours with respective treatments of Doxorubicin (750 nM or 2.5 μM) or 20 μM Roscovitine alone or in combination and graph representing average cell cycle distributions from three consecutive independent experiments. [file 1476-4598-9-208-S2.PDF]

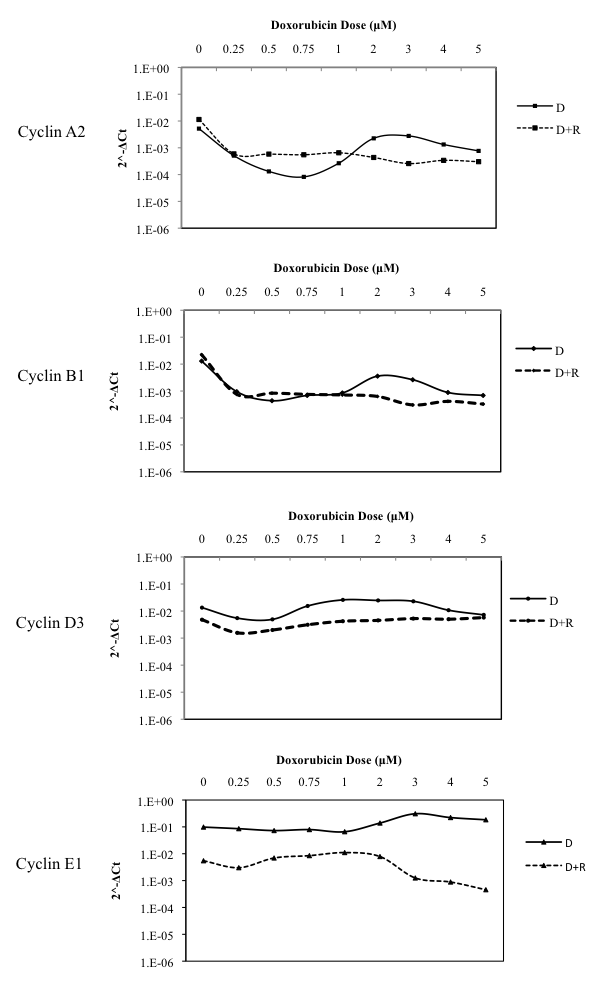

Supplement: Additional file 3 — Drug induced changed in cyclin mRNA expression levels. Expression levels respect to GAPDH (2^-ΔCt), in mRNA of cyclin A1, cyclin A2, cyclin B, cyclin D and cyclin E after 24 hours of treatment with either increasing doses of Doxorubicin (250 nM to 5 μM) alone or in combination with 20 μM Roscovitine. [file 1476-4598-9-208-S3.PDF]
