# Supplementary material for: R-Roscovitine (Seliciclib) prevents DNA damage-induced cyclin A1 upregulation and hinders non-homologous end-joining (NHEJ) DNA repair
Source: Mol Cancer. 2010 Aug 4;9:208. doi: 10.1186/1476-4598-9-208 (PMC3224749; doi:10.1186/1476-4598-9-208)
Supplement: Additional file 4 — Table of gene specific primer sequences utilized in this manuscript. [file 1476-4598-9-208-S4.PDF]

Gene Name	Forward Primer	Reverse Primer
**CCNA1**	5’-CTCCTCTCCCAGTCTGAAGA-3’	5’CAGGAAGTTGACAGCCAGAT-3’
**CCNA2**	5’-AATAGAGCGTGAAGATGCCCTGGC-3’	5’-GGGTGCAACCCGTCTCGTCTTCG-3’
**CCNB1**	5’-CCGAGTCACCAGGAACTCG-3’	5’-GCTGTTCTTGGCCTCAGTC-3’
**CCND3**	5’-CTCTGTGCTACAGATTATACC-3’	5’-GCCAGCAGCTCTGTGAGC-3’
**CCNE1**	5’-GAGGAAGGCAAACGTGACC-3’	5’-TGTCCCAAGGCTGGCTCC-3’
**GAPDH**	5’-GAAGGTGAAGGTCGGAGT-3’	5’-CATGGGTGGAATCATATTGGA-3’
**pCI-neo**	5’-GGCTGCTATTGGGCGAAGTG-3’	5’-GATGTTTCGCTTGGTGGTCG-3’
**pCI-neo BamHI**	5’-CAACCTGCCATCACGATGG-3’	5’-GCTTGTCTGTAAGCGGATGC-3’
